# Supplementary material for: Mechanistic insight into AP-endonuclease 1 cleavage of abasic sites at stalled replication fork mimics
Source: Nucleic Acids Res. 2023 Jun 2;51(13):6738–53. doi: 10.1093/nar/gkad481 (PMC10359615; doi:10.1093/nar/gkad481)
Supplement: gkad481_Supplemental_File [file gkad481_supplemental_file.pdf]

Supplemental Information for

Mechanistic Insight into AP-Endonuclease 1 Cleavage of Abasic Sites at Stalled Replication Fork Mimics

Nicole M. Hoitsma[1], Jessica Norris[2], Thu H. Khoang[1], Vikas Kaushik[3], Rahul Chadda[3], Edwin Antony[3], Mark Hedglin[2], and Bret D. Freudenthal[1]*

[1] Department of Biochemistry and Molecular Biology, University of Kansas Medical Center, Kansas City, KS, 66160, USA

[2] Department of Chemistry, The Pennsylvania State University, University Park, PA, 16802, USA

[3] Department of Biochemistry and Molecular Biology, Saint Louis University School of Medicine, St. Louis MO, 63104, USA

* To whom correspondence should be addressed. Tel: 913-588-5560; Email: bfreudenthal@kumc.edu

Supplemental Figures S1-S12

Supplemental Table S1

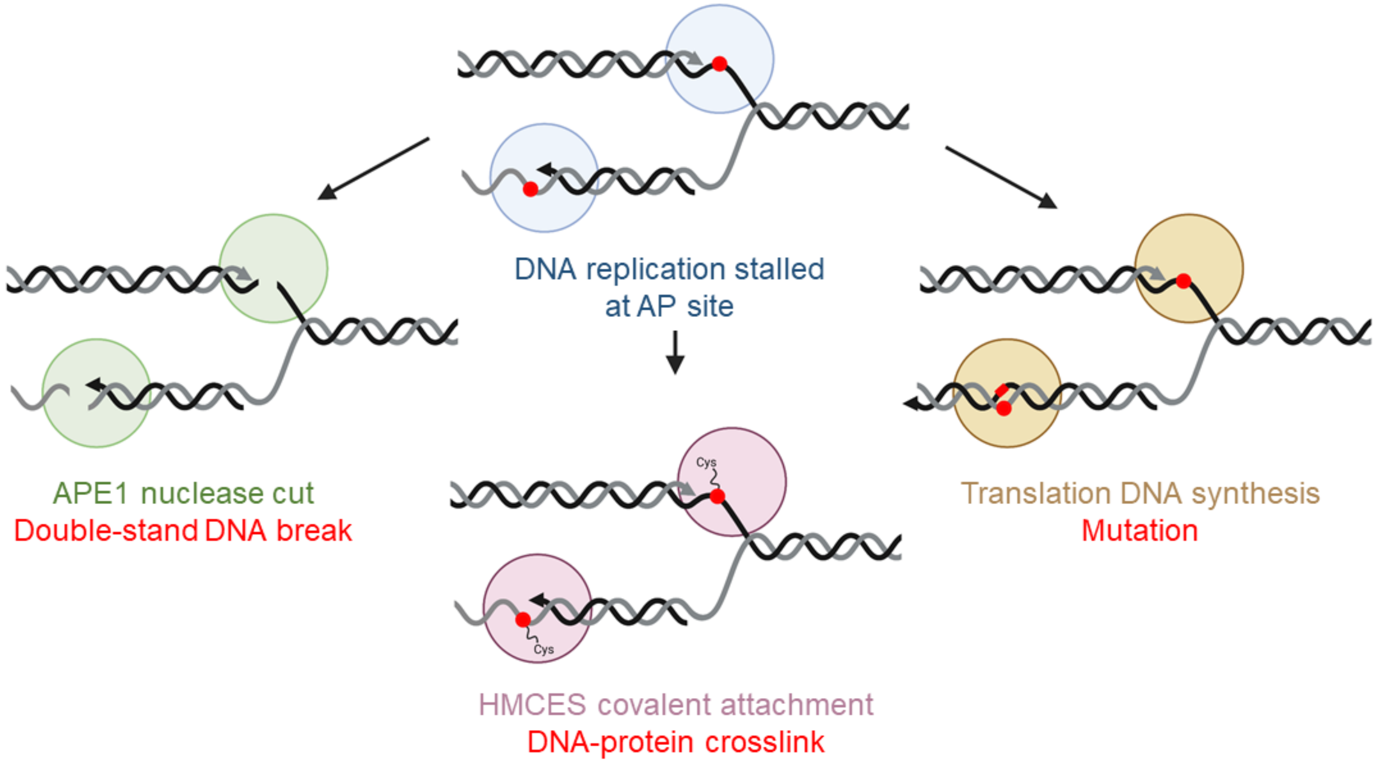

Figure S1. Pathways for AP site repair/bypass at a replication fork.

When the replicative DNA polymerase (blue, top) encounters a AP site (red sphere) in either leading or lagging strand synthesis, it will stall the replication fork and processing can occur via a number of pathways: (1) the abasic site can be cleaved by the nuclease APE1 (green, left) generating a double strand DNA break, (2) the abasic site can be covalently attached to the HMCES protein (pink, bottom) generating a DNA protein crosslink, or (3) the abasic site can be bypassed by a translesion DNA polymerase (yellow, right) generating mutations due to the lack of coding potential. We have not included all possible pathways to deal with a stalled replication fork and point the reader to a recent review discussing the various replication stress pathways [24]. Figure created with BioRender.com.

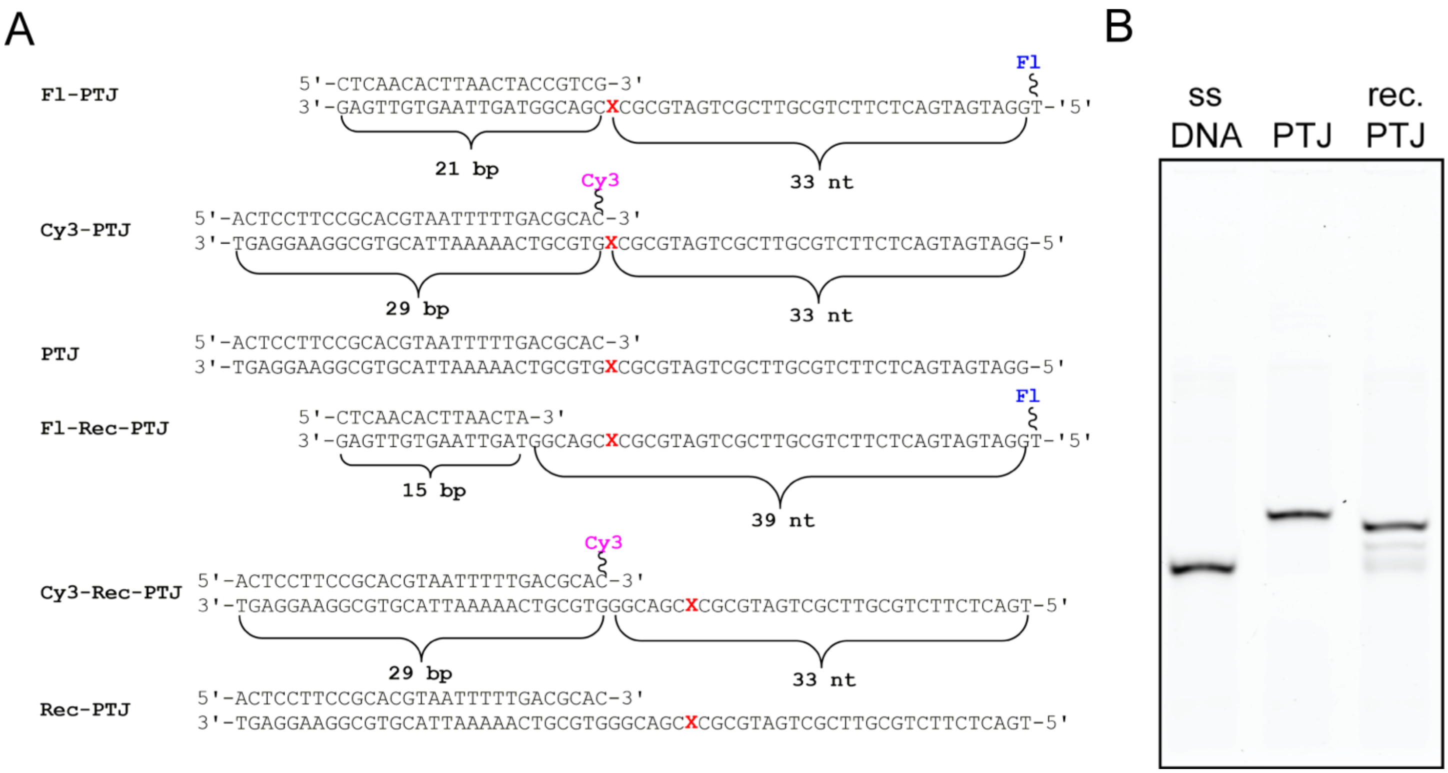

Figure S2. DNA substrates utilized in this study.

A) Primer-template junction substrates (PTJ). **X** denotes an abasic site analog (Tetrahydrofuran, THF). Cy3 denotes Cyanine 3. Fl denotes fluorescein. The ssDNA regions adjacent to the 3'-end of the Primer-Template junctions (PTJ) accommodate at least 1 RPA molecule.

(B) Native acrylamide gel of annealed fluorescein-containing DNA substrates, visualized by fluorescence of the fluorescein-labeled, 54-mer primer strand. Shown in lane 1 is free primer (i.e., ssDNA). Lanes 2 and 3 shown the primer annealed in the PTJ and Rec-PTJ DNA substrates, respectively.

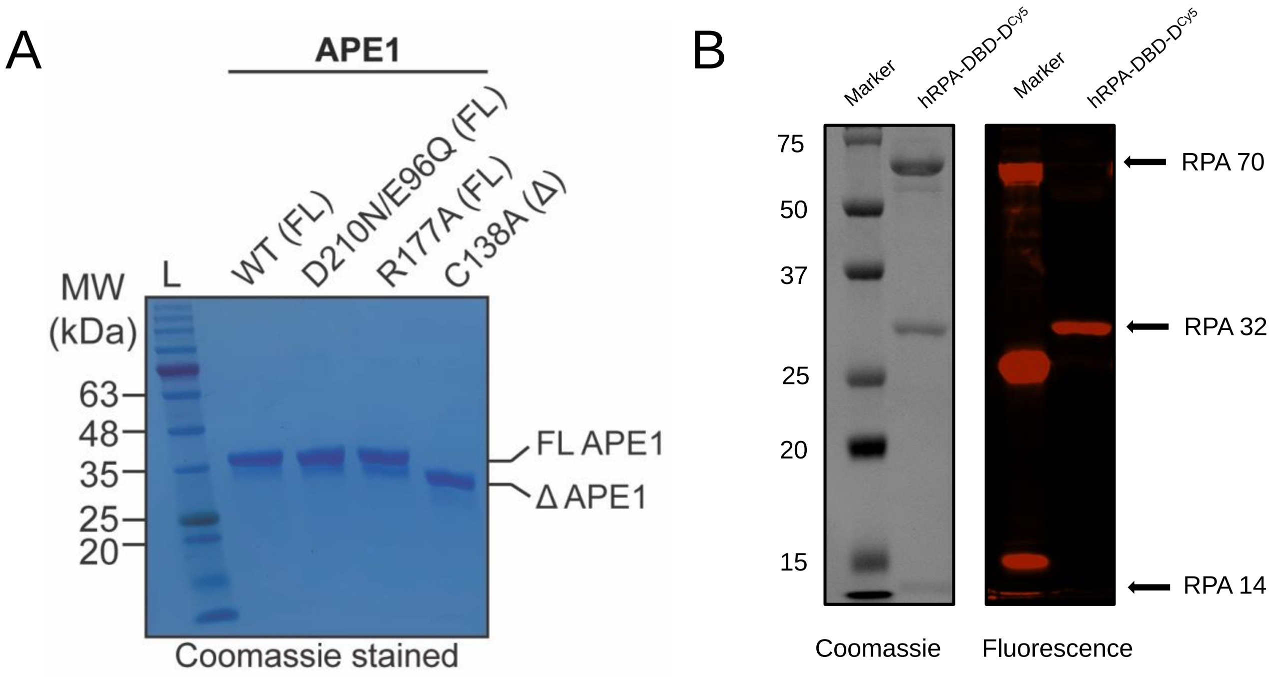

Figure S3. Purified recombinant proteins.

(A) Coomassie imaging for SDS PAGE gel of purified APE1 proteins used in this study.

(B) Coomassie and fluorescence imaging of human RPA labeled with Cy5. The protein is site-specifically labeled at position 107 in the RPA32 subunit. Correspondingly, only the labeled-RPA32 subunit is observed under fluorescence imaging (right panel).

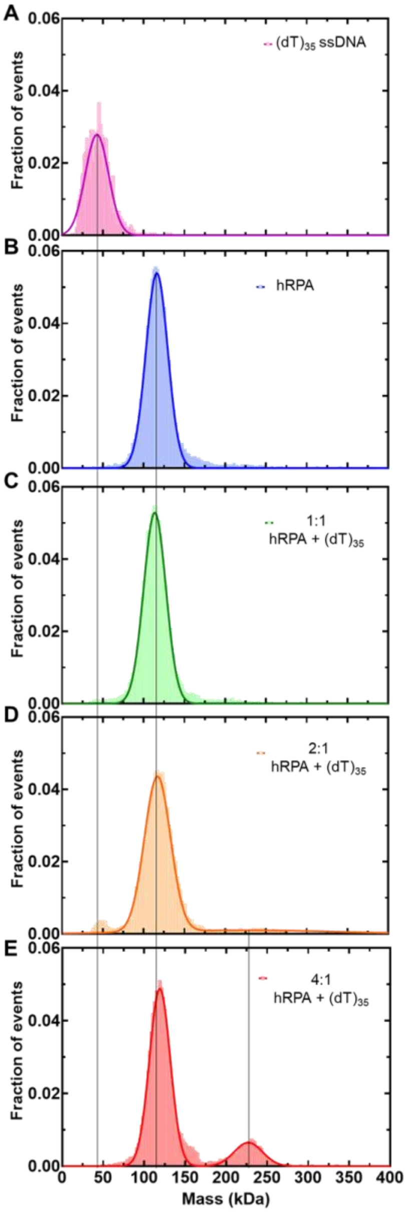

Figure S4. Mass photometry analysis of human RPA bound to $(dT)_{35}$.

Estimated molecular weight (M.W.) distribution of ssDNA alone (200 nM $(dT)_{35}$, panel **A**), human RPA alone (200 nM, panel **B**) and equilibrium complexes of human RPA and ssDNA (200 nM $(dT)_{35}$, 200 – 800 nM RPA, panels **C** – **E**). For equilibrium complexes, ssDNA (200 nM $(dT)_{35}$) was mixed with different concentrations of human RPA (200, 400, or 800 nM) resulting in 1:1, 1:2, and 1:4 DNA:RPA ratios. Mass photometry histograms were fitted with a Gaussian model. The kDa units in the plot corresponds to the respective mass at the center of the peak. Note that a second high M.W. peak is only observed at a 1:4 DNA:RPA ratio (panel **E**) and this peak accounts for only 15% of RPA:DNA complexes. $(dT)_{35}$ is comprised of a linear sequence of 35 thymidines and is devoid of secondary structure.

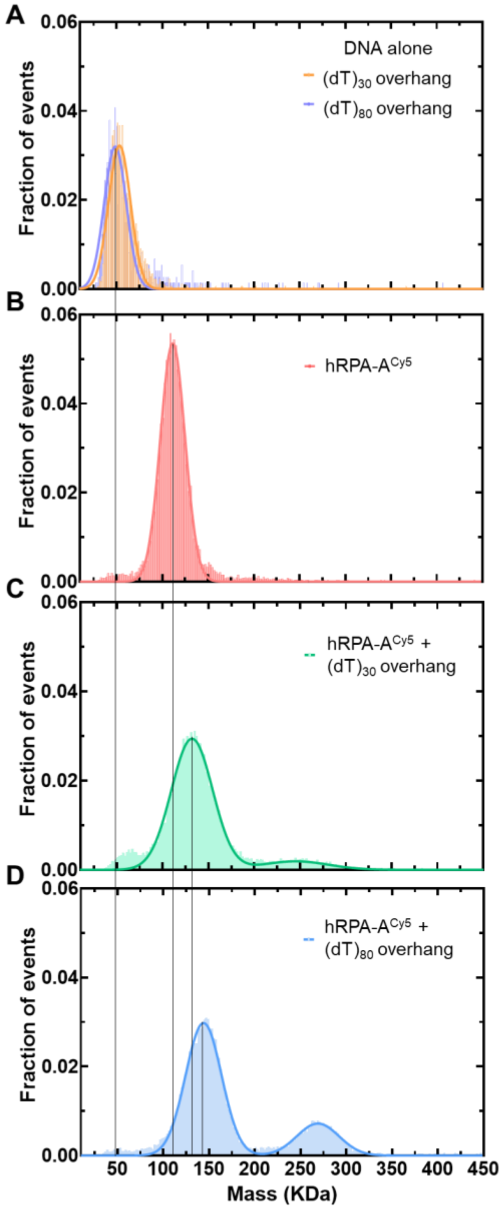

Figure S5: Mass photometry analysis of human RPA bound to PTJ DNA substrates with 5′ ssDNA overhangs of different lengths.

Estimated molecular weight (M.W.) distribution of PTJ DNA substrates alone (200 nM, panel **A**), human Cy5-RPA alone (200 nM, panel **B**) and equilibrium complexes of human RPA and a PTJ DNA substrate (200 nM DNA, 600 nM Cy5-RPA, panels **C** and **D**). PTJ substrates were comprised of a 20 bp duplex region, a 3′ recessed end labeled with a Cy3 and a 5′ ssDNA overhang comprised of either $(dT)_{30}$ or $(dT)_{80.}$ For equilibrium complexes, a PTJ DNA substrate (200 nM) was mixed with human Cy5-RPA (600 nM) resulting in 1:3 DNA:RPA ratio. Mass photometry histograms were fitted with a Gaussian model. The kDa units in the plot corresponds to the respective mass at the center of the peak. Note that a second high M.W. peak is observed for each PTJ DNA substrate, but this peak accounts for only 13% and 20% of all RPA:DNA complexes observed for the $(dT)_{30}$ and $(dT)_{80}$ overhang PTJ DNA substrates, respectively.

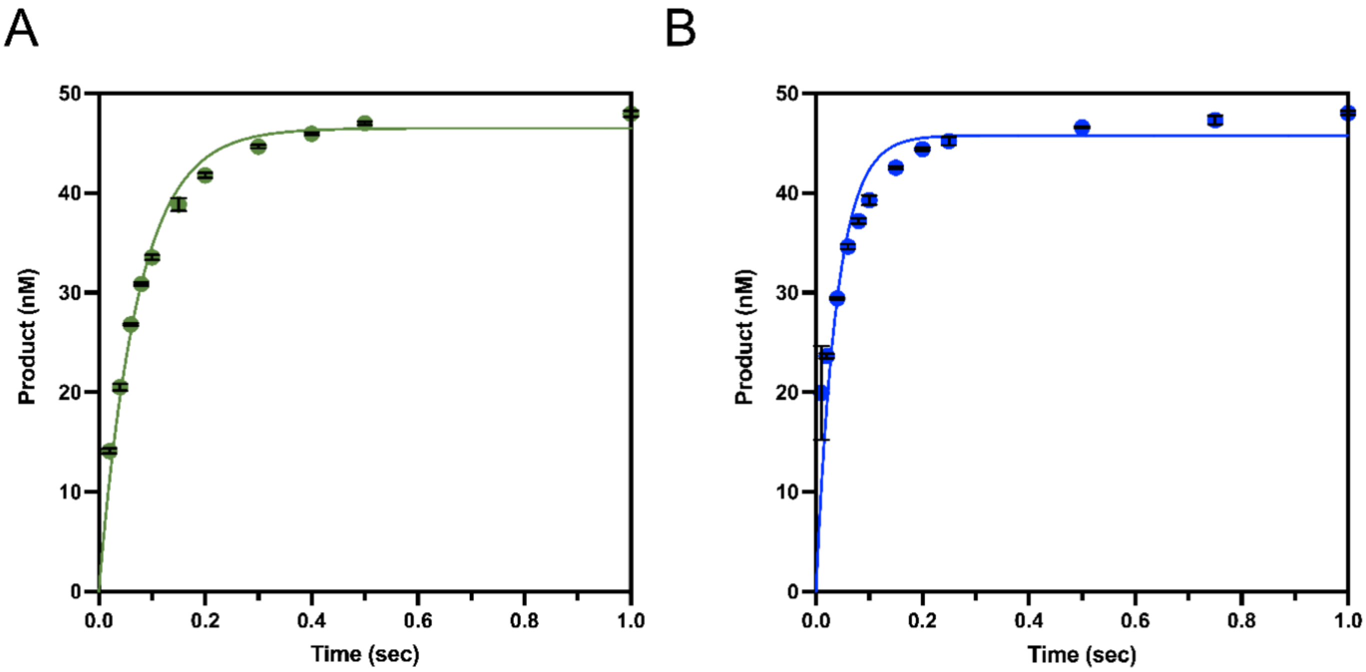

Figure S6. Single turnover kinetic time courses for WT APE1.

Single turnover kinetic time courses for WT APE1 (500 nM) with (**A**) PTJ (green) and (**B**) Rec-PTJ (blue) (DNA substrates at 50 nM). All time points are shown as the mean of three independent experiments with error bars (st. dev.) shown. Where error bars are not seen, they are smaller than the data point.

Table S1. Data collection and refinement statistics.

Highest resolution shell is shown in parentheses.

	Product Complex (AP-ssDNA)
**Data collection**	
Space group	P 65
Cell dimensions	
a, *b*, *c* (Å)	83.7, 83.7, 204.2
α, β, γ (°)	90.0, 90.0, 120.0
Resolution (Å)	25.00 – 2.00
R_{meas} (%)	0.080 (0.577)
$I/\sigma I$	20.9 (2.7)
cc1/2	(0.759)
Completeness (%)	100.0 (99.9)
Redundancy	6.3 (4.3)
**Refinement**	
Resolution (Å)	24.81 – 2.00
No. reflections	83056
$R_{work/}$ R_{free}	18.2/21.5
No. atoms	
Protein	4384
DNA	288
Water	356
B-factors (Å^2)	
Protein	28.20
DNA	30.46
Water	30.49
R.m.s deviations	
Bond length (Å)	0.011
Bond angles (º)	1.088
PDB ID	7TR7

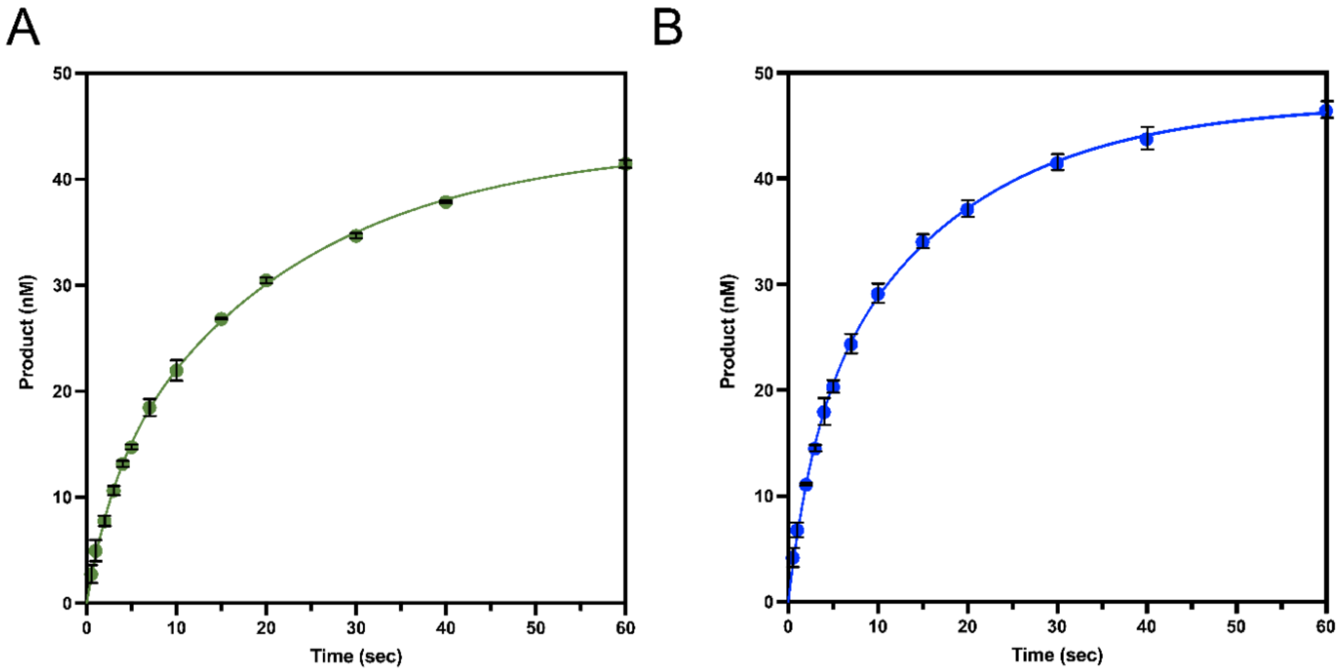

Figure S7. Single turnover kinetics with APE1 R177A.

Single turnover kinetic time courses for APE1 R177A (500 nM) with (**A**) PTJ (green) and (**B**) Recessed PTJ (blue) (DNA substrates at 50 nM). All time points are shown as the mean of three independent experiments with error bars (st. dev.) shown. Where error bars are not seen, they are smaller than the data point.

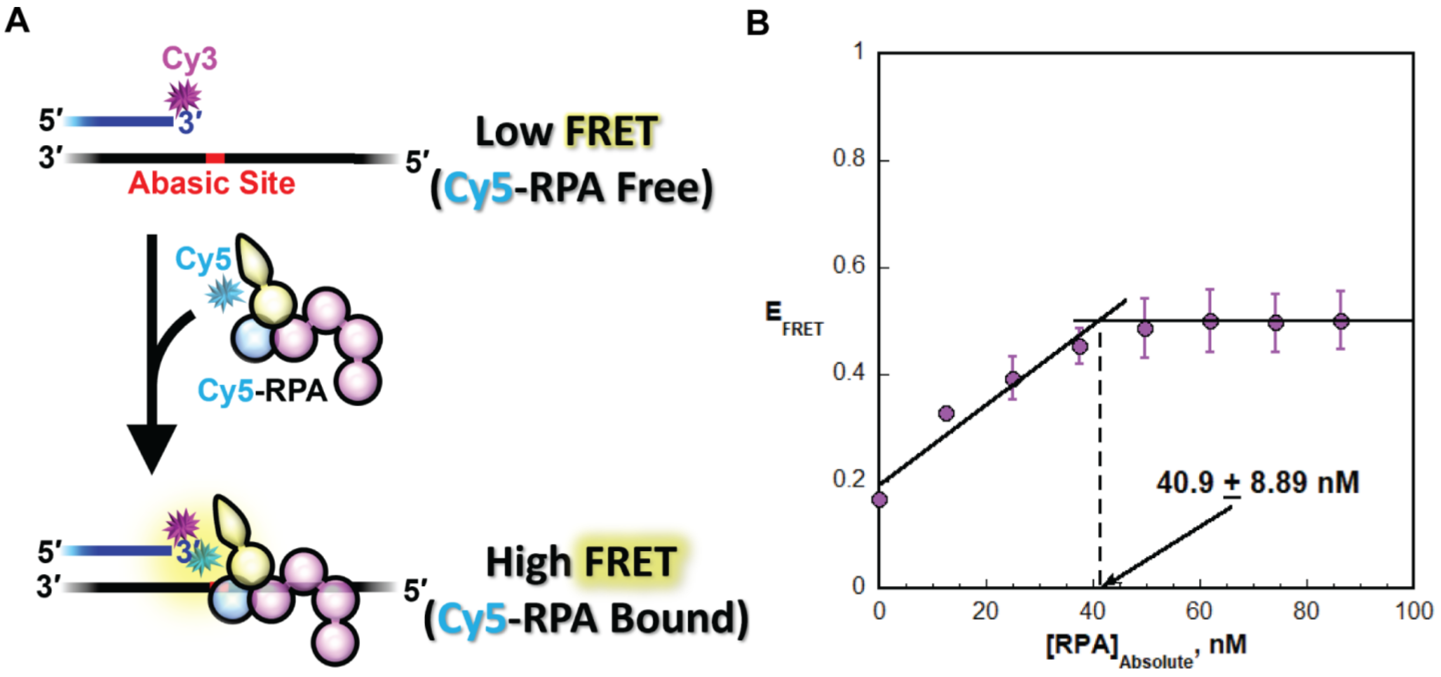

Figure S8. FRET-based titration of Rec-PTJ with Cy5-RPA.

The substrate for this assay is a Rec-PTJ (also depicted in **Figure S2A**) that contains a Cy3 (FRET donor) fluorophore at the 3′ terminus of the primer strand and an abasic site 7 nt downstream of the PTJ.

(**A**) Schematic representation of experimental procedure. In the absence of Cy5-RPA (FRET acceptor), a FRET is not observed. The ssDNA downstream of the PTJ (33 nt) can accommodate a single Cy5-RPA. RPA engages the ssDNA in an orientation-specific manner such that the Cy5-labeled DBD-D of the RPA32 subunit faces the Cy3 FRET donor on the PTJ, yielding a robust FRET. Hence, addition of Cy5-RPA increases FRET.

(**B**) FRET Data. Cy3-Rec-PTJ (25 nM) was titrated with Cy5-RPA and FRET is monitored. The observed E_{FRET} $[I_{665}/(I_{665} + I_{563})]$ is plotted as a function of the absolute concentration of added Cy5-RPA and each data point represents the mean ± S.E.M. of three independent measurements. Under these experimental conditions, binding is stoichiometric and, hence, FRET increases linearly until the ssDNA is saturated with Cy5-RPA (i.e., equivalence point). Data is fit to two segment lines (a linear regression with a positive slope and a flat line) and the equivalence point (indicated with standard error of the calculation) is calculated from the intersection of the two segment lines. Saturation is reached at 40.9 ± 8.89 nM Cy5-RPA.

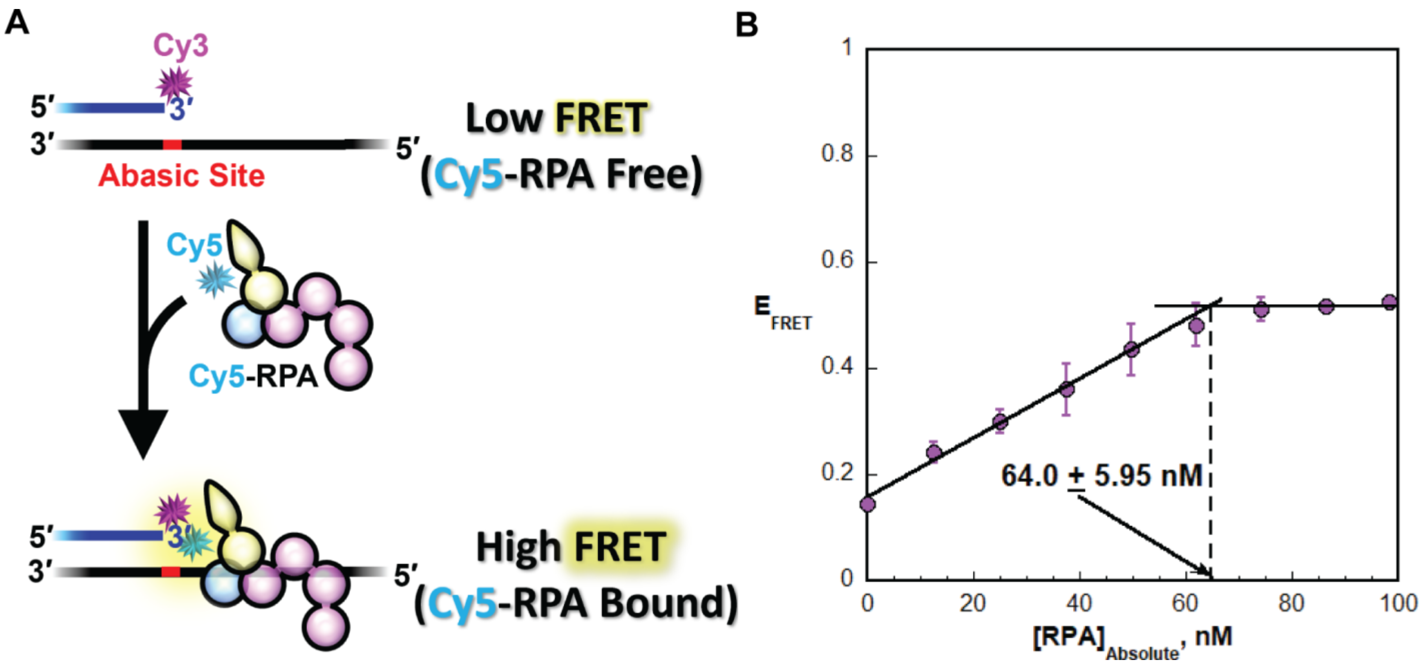

Figure S9. FRET-based titration of PTJ with Cy5-RPA.

The substrate for this assay is a PTJ (also depicted in **Figure S2A**) that contains a Cy3 (FRET donor) fluorophore at the 3′ terminus of the primer strand and an abasic site at the PTJ.

(**A**) Schematic representation of experimental procedure. In the absence of Cy5-RPA (FRET acceptor), a FRET is not observed. The ssDNA downstream of the PTJ (33 nt) can accommodate a single Cy5-RPA. RPA engages the ssDNA in an orientation-specific manner such that the Cy5-labeled DBD-D of the RPA32 subunit faces the Cy3 FRET donor on the PTJ, yielding a robust FRET. Hence, addition of Cy5-RPA increases FRET.

(**B**) FRET Data. The Cy3-PTJ substrate (25 nM) was titrated with Cy5-RPA and FRET is monitored. The observed E_{FRET} [$I_{665}/(I_{665} + I_{563})$] is plotted as a function of the absolute concentration of added Cy5-RPA and each data point represents the mean ± S.E.M. of three independent measurements. Under these experimental conditions, binding is stoichiometric and, hence, FRET increases linearly until the ssDNA is saturated with Cy5-RPA (i.e., equivalence point). Data is fit to two segment lines (a linear regression with a positive slope and a flat line) and the equivalence point (indicated with standard error of the calculation) is calculated from the intersection of the two segment lines. Saturation is reached at 64.0 + 5.95 nM Cy5-RPA added.

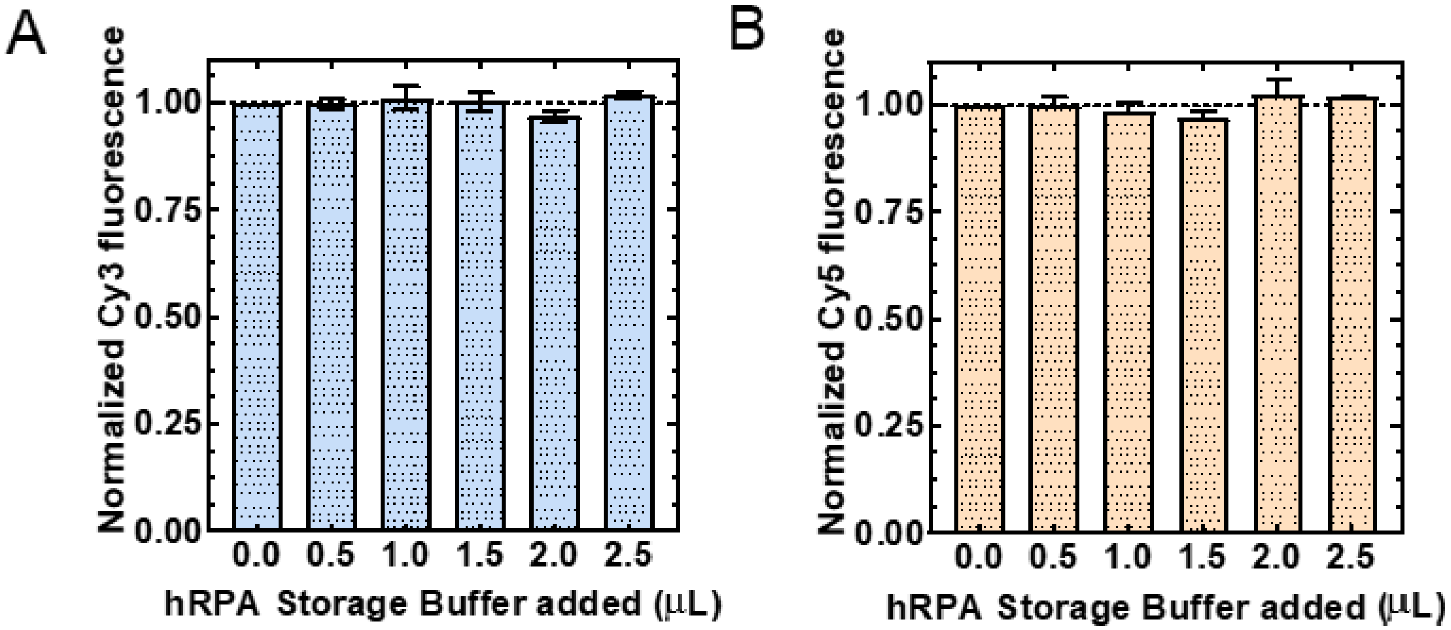

Figure S10. Buffer and dilution controls for Cy3 and Cy5 fluorescence.

(A) Effect of human RPA storage buffer on the fluorescence of Cy3-DNA. Human RPA storage buffer was added in 0.5 μL increments to 25 nM Cy3-DNA taken in 120 μL reaction mixture as the sample was excited with 514 nm light and the fluorescence emission at 563 nm (I_{563}) is recorded. I_{563} values were not corrected for by any dilution factor. I_{563} values are normalized to the initial value (i.e., zero dilution) and the mean and standard deviation from three trials are plotted as a function of the total volume of added buffer. The Cy3 fluorescence shows little systematic change as a function of added buffer volume.

(B). Effect of human RPA storage buffer on the fluorescence of Cy5-RPA. Human RPA storage buffer was added in 0.5 μL increments to 78 nM Cy5-RPA taken in 120 μL reaction mixture as the sample was excited with 635 nm light and the fluorescence emission at 665 nm (I_{665}) is recorded. I_{665} values were not corrected for by any dilution factor. I_{665} values are normalized to the initial value (i.e., zero dilution) and the mean and standard deviation from three trials are plotted as a function of the total volume of added buffer. The Cy5 fluorescence shows little systematic change as a function of added buffer volume.

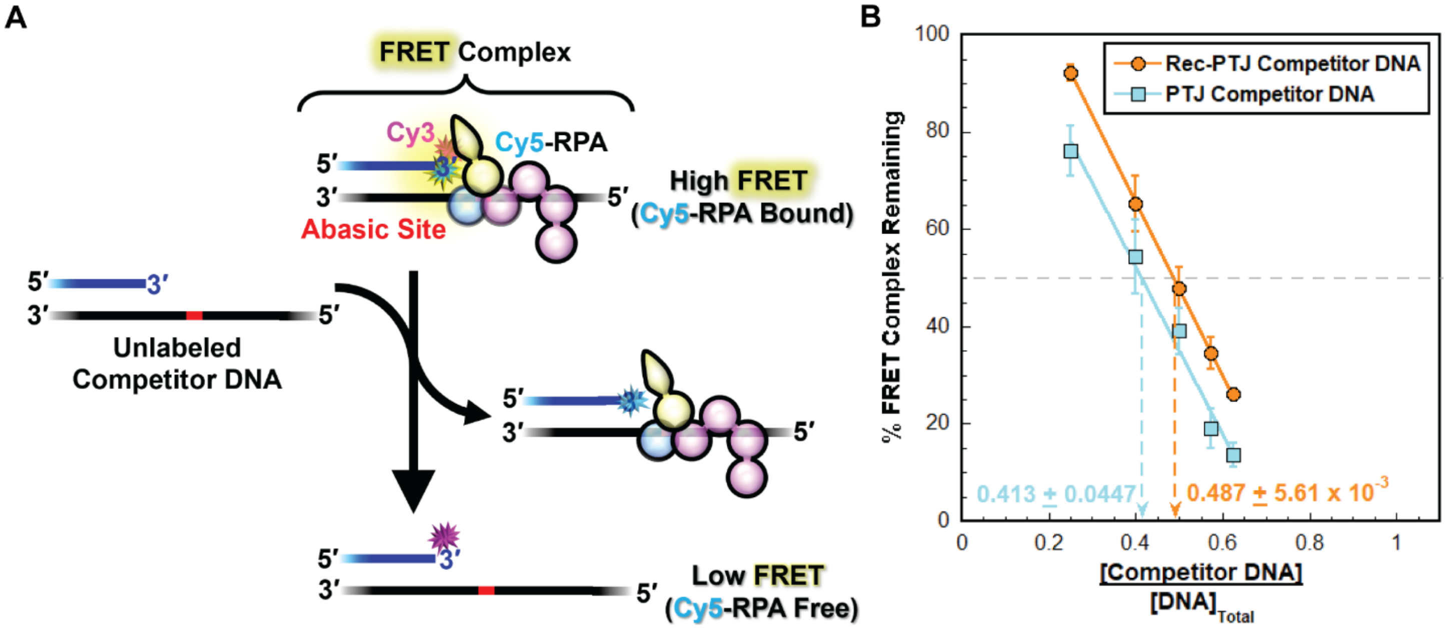

Figure S11. RPA exchange on PTJ DNA containing abasic sites at different positions.

(**A**) Schematic representation of the FRET experiment. A 3′ Cy3-labeled Rec-PTJ DNA (25 nM) is pre-saturated with Cy5-labeled RPA (75 nM absolute) and the resultant mixture is then titrated with the respective unlabeled PTJ competitor DNA. E_{FRET} is calculated after each addition of competitor.

(**B**) Observed E_{FRET} values for a given competitor DNA are normalized to their respective range and plotted as a function of [Competitor DNA]/[DNA]$_{Total}$ and each data point represents the mean ± S.E.M. of at least three independent measurements. The values observed after the addition of competitor DNA are fit to a linear regression. The [Competitor DNA]/[DNA]$_{Total}$ required for 50% inhibition (i.e., the % FRET Complex remaining decreases to 50%) is calculated from the fit and reported for each competitor DNA.

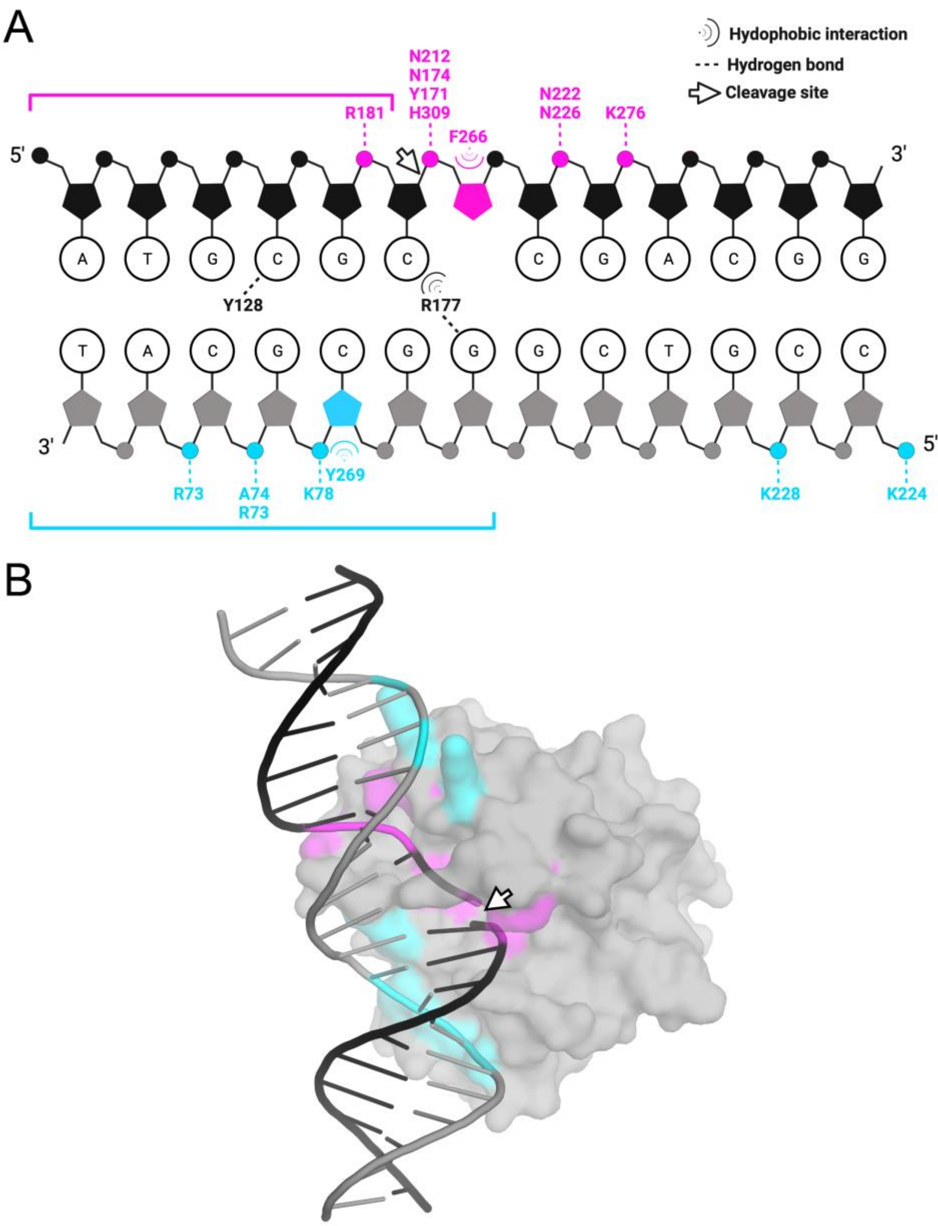

Figure S12. APE1 interactions with abasic dsDNA.

(**A**) Schematic of the interactions between wild-type APE1 and abasic dsDNA. Interactions with abasic and opposing nondamaged strands are shown in pink and cyan, respectively. The pink bracket highlights the 5’ downstream region of the abasic strand that is absent from the APE1:ssDNA product complex crystal structure. The cyan bracket highlights the region of the nondamaged strand that would be absent in a PTJ structure. Interactions were determined using the protein–ligand interaction profiler, PLIP, available at https://plip-tool.biotec.tu-dresden.de with hydrogen bonds limited at 3.8 Å [84]. Figure created with BioRender.com.

(**B**) Overview of APE1:AP-dsDNA structure with APE1 (grey, surface) bound to dsDNA (black and grey, cartoon). The DNA and interacting residues (as shown in panel **A**) are highlighted in pink or cyan, based on interaction with the abasic or opposing nondamaged strand, respectively. Cleavage site is denoted with a white arrow (PDB 5DFF).
